# Clinical Lecture on the Treatment of Pneumonia, Delivered at St. Mary’s Hospital, May 10th, 1862

**Published:** 1863-03

**Authors:** Thos. K. Chambers

**Affiliations:** Lecturer on Clinical Medicine


### CLINICAL LECTURE ON THE TREATMENT OF PNEUMONIA.

DELIVERED AT ST. MARY'S HOSPITAL, MAY 10, 1862.

By THOS. K. CHAMBERS, M. D., Lecturer on Clinical Medicine.

GENTLEMEN: There are three cases of pneumonia under my care in Albert ward this week, to which I have drawn your attention as illustrative of those of the most common phases under which you have to treat that disease in the adult. No. 1. Frank, uncomplicated, double pneumonia, in a temperate, robust man; excessive dyspnœa. Cured by venesection, jacket poultice, food every two hours, wine. No. 2. Pneumonia of upper and lower lobes of right lung, very slight in left lung, in a broken down old man. Cured by cupping in front, jacket poultice, food every two hours, and wine. No. 3. Congestive pneumonia of one lower lobe in typh fever. Cured by half-jacket poultice, cupping below scapula, food every two hours, wine, and bark.

(*The details of the cases are omitted here to save space.*)

In pneumonia a terribly vital organ is smitten, and so far as the disease extends the destruction is total. A consolidated or even congested piece of pulmonary tissue is impotent to fulfil its functions, and yet that those functions should be fulfilled is essential to animal existence. It is easy therefore to see that the gravity of the pneumonia is in a direct ratio to the quantity of lung involved. The degree or form of the inflammation or condensation is of much less moment, so far as immediate danger is concerned, than the extent of tissue over which it is spread.

Hence comes the importance of having some ready and effectual means at hand of checking the march of the inflammation to fresh parts. If we can do this, we contribute more certainly to the patient's life than if we regulate, however favorably, the progress of it in already affected places. No means is so readily applied, so immediate in its operation, as bloodletting. Its action has not to be waited for, like that of drugs in medicinal doses, but begins at the moment of application. That is a great point where time is so valuable. I believe also that it is the most powerfully effectual of the agents at our disposal, and that, rightly used, it is the saving of many a life in pneumonia.

The beneficial action of bloodletting in pneumonia is mechanical. It is more a question of hydrostatics than of physiology. The pathology of pneumonia is as follows: By the temporary death of a portion of the lungs, the blood cannot be quickly enough passed onwards through their tissue; it can run freely as far as the right side of the heart, but there it is stopped; the throng pressing onwards from behind makes matters worse, and thus the balance between the venous and arterial heart is destroyed. You can feel the apex of the organ beating strongly against the ribs, the muscular action being excited by the presence of an unusual amount of venous blood; yet the artery at the wrist is at the same time striking your finger with an imperfect, weakened force.

Take away some of the blood from the veins, and the balance is restored—the pulse becomes "freer," as the technical phrase is; that is to say, the heart being relieved of the undue crowd in the right side, is not arrested in its contraction, but is able to close upon its contents, and supply them steadily to the arteries.

The advantage of general and local bloodletting is of identically the same nature, though they differ somewhat in degree, and are diversely applicable. Where the patient was, previous to his current illness, in vigorous health, actively digesting his food, and actively renewing his tissues, he will bear, and easily repair, the detraction of a good large quantity of blood; and a good large quantity of blood is most conveniently drawn from the arm. To get, therefore, the full advantage of the remedy, and be on the safe side, you should practice venesection. But if the pneumonia has come on a person previously an invalid, or in weak health, you fear for the possible bad consequences of your treatment, and you cast about for some means of getting the greatest advantage out of the least loss of blood. This is obtained by cupping the region of the heart. Your six or seven ounces taken from thence, in a delicate invalid, seem to produce a corresponding effect to twelve or fourteen let from a vigorous man's arm. But there are practical inconveniences in cupping to a large amount in this situation. You are obliged to cut deep to obtain a good flow, and deep cuts cannot be stopped easily, but go on oozing unperceived into the poultice which, as I will instruct you presently, is to be put round the chest.

Remember that in letting blood you are wielding a dangerous weapon. While from a mechanical point of view nothing can equal the aid it gives, at the same time its more remote or physiological action is hurtful. The shrewd comedian tells us, "necesse est facere sumptum, si quæris lucrum;" so that if you have gained the inestimable boon of a restoration of balance in the circulation, and a consequent relief of dyspnœa and an arrest of the progress of death in the lungs, you must not complain if some evils attend the process. The mere loss of so much "liquid flesh" is in itself an evil, but a minor one; of greater import is the increased proportion of effete fibrin and water which it induces, the diminution of red globules, and the consequently diminished power to bear up against the destruction, however temporary, of so much pulmonary substance.

Judge, therefore, of the necessity for this treatment by the balance between the heart and the arteries. If the apex of the former organ strikes strong while the pulse at the wrist is defective, act freely and confidently. If, on the contrary, the ventricles are weak while the pulse is full, large, and rapping, be cautious in what you do, and if you draw blood at all, let it be by cupping the chest.

You will find in some lectures on medicine rules about bloodletting in pneumonia attempted to be deduced from the supposed degree of consolidation of the pulmonary tissue. These rules are singularly foolish and inapplicable to practice. They say you should bleed so long as you know that the lung is in its first stage of consolidation, (*i. e.*, congestion), as indicated by fine crepitation and incomplete dulness; and that you should not bleed after it has once become so completely consolidated as to admit no air into the finer bronchi—a state declared by the sound of coarse crepitation and complete dulness. Such a rule is quite useless at the bedside, and will often prevent your employing active practice in cases where it is urgently required. In the first place, in a majority of cases fine crepitation is masked by the mixture of coarse, produced by the presence of catarrhal mucus in the larger bronchi, especially in the catarrhal pneumonia of young persons. If you wait till you can hear distinctly fine crepitation, you will wait too long. Then, again, the dulness of congestion is not necessarily incomplete, as you may satisfy yourselves by examining a case of transitory congestion in continued fever, which is often very absolute, though it is so transitory that a mere change of position may remove it in twenty-four hours. Then, again, a slight collection of serum in the pleura may make the lower lobe dull at the very commencement of pneumonia, and prevent your bleeding at a very early stage, if you were to follow the rule I quoted. But the most truly important consideration and the most serious objection to the rule is, that you may have all stages of partial tissue-death going on at the same time: one lobe, or one part of a lobe, may have advanced even to yellow hepatization, while another part is entering into red hepatization, and in a condition which most would agree is that capable of benefit from letting blood. Your best guide to the necessity will be the dyspnœa, and your best check, the balance of the heart and arteries, as I have explained already.

Remember now what I told you about bleeding in a former lecture on "Anæmia and Bloodletting"—*be careful to supply material in the place of that which you are taking away.* Let the patient be fed with beef-tea or milk every two hours, just as if he had typh fever. I mention this part of the treatment next to the bleeding to remind you of the close connection which there is between the two, and because of the immense importance of it to your success, whether you elect to bleed or do not.

I now come to a direct restorative, about the use of which at all times you need have no manner of hesitation. You can always, without any exception of age, sex, condition, cause, or complication, follow a treatment to which I attribute more power of saving the lives of pneumonic patients than to any other, and which you see me apply in all cases; I mean, the enveloping the chest in a large bath-like poultice. The action of warmth and moisture on animal tissues tends directly to increase their vitality. You may *see* with the naked eye a healthy surface of skin under their application renew its life; it empties itself quicker of its pale, livid, venous blood, and glows with a fresh access of the bright arterial stream; it swells up elastically with fresh juices; it is more delicately sensitive when used for the purposes of touch; at the same time it feels no pain, but, on the contrary, an exquisitely pleasurable calm. You cannot see this renewal of life in internal organs; but you may infer that what takes place in one tissue takes place also in another with modifications dependent on distance and other difficulties of application. And you may infer it also from the results; for you find the dyspnœa diminished, the breath being easier drawn in spite of the weight of the poultice; the hot, fevered skin becomes moist and active, and soon the ribs begin to move again and air is readmitted into the previously paralyzed lung-tissue. These effects are the most strikingly shown in the case of infants, whose thin chest-walls are rapidly and efficiently permeated by the influences of the poultice, and in whom also this remedy is the only one really safe and invariably necessary. I cannot speak too strongly of the importance of your adopting it, and letting all other treatment be rather rejected than this directly restorative agent.

The poultice is best made of bruised meal, because that keeps moistest. It should be spread half an inch thick at least, on a cloth or flannel, as broad as the circumference of the thorax. If any portion of the upper lobes is inflamed it is essential, and even if only the lower lobes are inflamed it is prudent, that it should be deep enough to cover the whole chest from the collar bones to the hypochondria. Lay the patient on it on his back, and fold it across the front until it meets. In adults it will usually keep in its place of its own accord; but in children it is useful to have a tape stitched on in front and a tape behind, which you tie over each shoulder in the manner of a shoulder strap, otherwise the little prisoners wriggle out of their soft breastplates. When once you have got it *in situ*, keep it there, and desire the nurse, on pain of dismissal, never to take it off till another hot one is ready to go on.

In low fever the continuous poultice somewhat stands in the way of the cool sponging. But, in practice, this last important part of the treatment becomes less necessary at the period when congestion and pneumonia occur; the skin has then become cooler and more active. Besides, the poultice often takes its place by softening and suffusing with a gentle perspiration the whole body. I have often had pneumonic patients complain of the way in which it makes them sweat.

Alcohol, especially in the form of port wine, is very useful in treating pneumonia. Even in hearty temperate persons, when you are going to bleed, it is desirable to give a little, as was done in Case 1. A glass of hot negus before the operation makes it safer; and whenever you observe the nervous system prostrated by the extent of the disease, so as to produce tremor of the hands, quivering of the tongue, delirium, dry brown tongue or a tendency thereto, throw in a little wine from time to time. In old persons, especially in the upper classes, who have been used to good living, and in persons of all ages who have indulged too freely in alcoholic liquids (like Case 2), you need not wait for any symptoms as above described, but begin with wine immediately. In children it is not required, and they get well quicker without it.

In the pneumonia of typh fever, position is of great importance. As long as the walls of bloodvessels retain their natural elasticity, they are able to resist the gravitating force which acts, of course, on the blood as on all matter; but when their life is lowered in disease, the elasticity is the first vital property which suffers, and the blood then gravitates towards the lowest part of the viscus. This is especially the case in typh fever. Lay the patient, therefore, on the side opposite to that affected (as was done in No. 3), or even on his face for a time, if both are affected; and thus the very force of gravitation, which you feared as an enemy, becomes a friend, by withdrawing the congestion from the weaker point. This boy was cupped on the side. You need not be afraid of a small loss of blood in typh fever, where an important viscus requires it. A large portion of the vital fluid you take away is poisoned and dead already, and unfit for the purposes of life; so that you are not robbing the patient to the full extent of the quantity drawn. You saw this lad was much more lively after his cupping than before. It is better to draw it locally than generally, because local benefit is expected from it, and not general.

I always abstain from giving purgatives in pneumonia. My reason is, because I have observed that patients who have diarrhœa at the same time generally do very badly; and if natural diarrhœa does harm, I infer that artificial diarrhœa does harm also. I prefer to produce constipation by opiates, where it does not already exist. If the rectum gets blocked up with feces, it is easy to wash it out with warm gruel.

Blisters, also, have seemed to me to do harm in a few cases where I have seen them employed before the patient came under my treatment. It is usually non-medical persons who put them on, under the general idea that they are good for a cough with pain in the chest.

Nothing has been said about antimony and mercury, drugs formerly much used in pneumonia. They are destructives, and I cannot see that there is anything to be destroyed in this disease, or that there is anything whose destruction would aid the employment of direct restorative treatment. When I used them, I was frequently obliged to leave them off on account of bad symptoms attributable to their agency, and I always felt doubtful if success in prosperous cases could be traced to them. But in all diseases which have been under treatment before yours, pray never let a word escape your lips, or a thought dwell upon your minds, about the patient being worse for the means previously employed. Most probably the harm done even by the most unsuitable drug is inappreciable; for a sick man is a tougher animal than we often give him credit for, and will stand a vast deal of faulty physic, and it can hardly be but what some of the treatment has added to his chances of life more than if he had been let alone. Besides, we are all infinitely fallible, God knows, and it is not for us to judge of circumstances we have not seen.—*Lancet*, Aug. 16, 1862.

